# Supplementary material for: Intermittent PI3Kδ inhibition sustains anti-tumour immunity and curbs irAEs
Source: Nature. 2022 May 4;605(7911):741–6. doi: 10.1038/s41586-022-04685-2 (PMC9132770; doi:10.1038/s41586-022-04685-2)
Supplement: Supplementary file 1 — Reporting Summary [file 41586_2022_4685_MOESM1_ESM.pdf]

nature research

Corresponding author(s): Christian H Ottensmeier
Last updated by author(s): Mar 1, 2022

Reporting Summary

Nature Research wishes to improve the reproducibility of the work that we publish. This form provides structure for consistency and transparency in reporting. For further information on Nature Research policies, see Authors & Referees and the Editorial Policy Checklist.

Statistics

For all statistical analyses, confirm that the following items are present in the figure legend, table legend, main text, or Methods section.

n/a	Confirmed	
☐	☒	The exact sample size (n) for each experimental group/condition, given as a discrete number and unit of measurement
☐	☒	A statement on whether measurements were taken from distinct samples or whether the same sample was measured repeatedly
☐	☒	The statistical test(s) used AND whether they are one- or two-sided<br>*Only common tests should be described solely by name; describe more complex techniques in the Methods section.*
☒	☐	A description of all covariates tested
☐	☒	A description of any assumptions or corrections, such as tests of normality and adjustment for multiple comparisons
☐	☒	A full description of the statistical parameters including central tendency (e.g. means) or other basic estimates (e.g. regression coefficient) AND variation (e.g. standard deviation) or associated estimates of uncertainty (e.g. confidence intervals)
☐	☒	For null hypothesis testing, the test statistic (e.g. F, t, r) with confidence intervals, effect sizes, degrees of freedom and P value noted<br>*Give P values as exact values whenever suitable.*
☒	☐	For Bayesian analysis, information on the choice of priors and Markov chain Monte Carlo settings
☒	☐	For hierarchical and complex designs, identification of the appropriate level for tests and full reporting of outcomes
☒	☐	Estimates of effect sizes (e.g. Cohen's d, Pearson's r), indicating how they were calculated

Our web collection on statistics for biologists contains articles on many of the points above.

Software and code

Policy information about availability of computer code

Data collection	FACS Diva 9.0
Data analysis	GraphPad Prism v9.02, Flowjo v10.4.1, R 3.5.0, Seurat (v3.1.5), DESeq2 (v1.24.0) TopHat (v2.09) for human data, TopHat (v1.4.1) for murine data, FastQC (v0.11.2), Bowtie (v.1.1.2), Samtools (v0.1.19.0), HTSeq framework (v0.7.1), Trimmomatic (v.0.36), ggplot2(v3.3.2), data.table (v1.13.2), RColorBrewer (v1.1.2), MAST (v1.10.0), SCDE (v1.99.1), python (v3.8.5), graphviz (v0.8.2), fgsea (v1.10.1), Cell Ranger (v3.1.0), bcl2fastq (v2.20.0.422), uwot (v0.1.8), cowplot (v1.0.0)

For manuscripts utilizing custom algorithms or software that are central to the research but not yet described in published literature, software must be made available to editors/reviewers. We strongly encourage code deposition in a community repository (e.g. GitHub). See the Nature Research guidelines for submitting code & software for further information.

Data

Policy information about availability of data

All manuscripts must include a data availability statement. This statement should provide the following information, where applicable:
- Accession codes, unique identifiers, or web links for publicly available datasets
- A list of figures that have associated raw data
- A description of any restrictions on data availability

Raw data are publicly available and GEO links are provided.

Field-specific reporting

Please select the one below that is the best fit for your research. If you are not sure, read the appropriate sections before making your selection.

☒ Life sciences ☐ Behavioural & social sciences ☐ Ecological, evolutionary & environmental sciences

For a reference copy of the document with all sections, see nature.com/documents/nr-reporting-summary-flat.pdf

Life sciences study design

All studies must disclose on these points even when the disclosure is negative.

Sample size

Murine studies: Sample sizes were chosen based on published studies to ensure sufficient numbers of mice in each group enabling reliable statistical testing and accounting for variability. Sample sizes are indicated in Figure legends. Clinical study: The sample size was calculated as follows: in a pilot cohort, the CD8 count in the biopsy taken at diagnosis, and in the resected tissue sample was quantified. The mean value at diagnosis was 25 cells/high power filed (hpf), and this remained almost the same in the resected sample (26 cells/hpf). With an observed standard deviation of five cells we posited we would observe a doubling to 50 cells/hpf following treatment with AMG 319, hence a difference between the two treatment groups of 25. To detect a standardised difference of 0.5 with 80% power and one sided test of statistical significance of 20%, we required 36 patients to be randomised to AMG319 and 18 to placebo (54 in total).

Data exclusions

RNA-seq samples that didn't pass quality control weren't included in the analyses.

Replication

Murine data were reliably reproduced in independent experiments at least twice.

Randomization

Murine studies: Animals of same sex and age were randomly assigned to experimental groups. Clinical study: Randomisation was at the level of the individual patient, using block randomisation with randomly varying block sizes. During the course of the clinical trial the randomisation list was held by the unblinded Trial Statistician and within the IWRS

Blinding

Murine studies: Blinding was not performed. The employed methods involve unbiased quantification (flow cytometry, gene expression). The experimental obervation (change in tumor volume) following therapeutic intervention was substantial and consistent within the respective groups. Hence, the data presented did not require blinding. Clinical study: During the course of the clinical trial the randomisation list was held by the unblinded Trial Statistician and within the IWRS. Patients and care providers were blinded to the treatment allocation, and all immunological evaluations were completed by a pathologist and researchers who were blinded to the patient allocation to treatment arms.

Reporting for specific materials, systems and methods

We require information from authors about some types of materials, experimental systems and methods used in many studies. Here, indicate whether each material, system or method listed is relevant to your study. If you are not sure if a list item applies to your research, read the appropriate section before selecting a response.

Materials & experimental systems

n/a	Involved in the study
☐	☒ Antibodies
☐	☒ Eukaryotic cell lines
☒	☐ Palaeontology
☐	☒ Animals and other organisms
☐	☒ Human research participants
☐	☒ Clinical data

Methods

n/a	Involved in the study
☒	☐ ChIP-seq
☐	☒ Flow cytometry
☒	☐ MRI-based neuroimaging

Antibodies

Antibodies used

The following antibodies for floytometry were used in this study.

Fixable Viability Dye, ThermoFisher Scientific, eFluor780

anti-human

Antibody, Supplier, Clone, Color

PD-1, BD Biosciences, EH12.1, BV421, 1:30, cat#562516
CD45, BD Biosciences, HI30, APC-R700, 1:30, cat#566041
CD25, BD Biosciences, M-A251, PE, 1:20, cat#555432
CD127, eBioscience, eBioRDR5, APC, 1:50, cat#17-1278-42
CD4, Biolegend, OKT4, BV510, 1:30, cat#317444
CD137, Biolegend, 4B4-1, BV605, 1:30, cat#309822

GITR, Biolegend, 108-17, BV711, 1:30, cat#371212
ICOS, Biolegend, C398.4A, BV786, 1:50, cat#313534
CD8A, Biolegend, SK1, PerCP-Cy5.5, 1:30, cat#344710
CD3, Biolegend, SK7, PE-Dazzle594, 1:30, cat#344844
CD14, Biolegend, HCD14, APC-Cy7, 1:50, cat#325620
CD20, Biolegend, 2H7, APC-Cy7, 1:50, cat#302314

anti mouse
ST2, BD Biosciences, U29-93, BV421, 1:100, cat#145309
CD8, BD Biosciences, 53-6.7, BB700, 1:100, cat#566409
Ki67, BD Biosciences, B56, BV786, 1:40, cat#563756
CD45, BD Biosciences, 30-F11, APC-R700, 1:100, cat#565478
TOX, Miltenyi, REA473, APC, 1:40, cat#130-118-335
FOXP3, eBioscience, FJK-16s, APC, 1:100, cat#17-5773-82
CD3, Biolegend, 145-2C11, BV510, 1:100, cat#100353
CD4, Biolegend, RM4-5, PE-Dazzle594, 1:100, cat#100566
PD-1, Biolegend, 29F1.A12, BV605, 1:100, cat#135220
CD19, Biolegend, 6D5, BV650, 1:100, cat#115541
GzmB, Biolegend, QA16A02, PE, 1:40, cat#372208

Validation

All anti-human and anti-mouse antibodies have been validated by the respective manufacturer for flow cytometry.

Eukaryotic cell lines

Policy information about cell lines

Cell line source(s)

B16F10-OVA cells were a gift from the laboratory of Prof. Linden (LJI)

Authentication

B16F10-OVA cells form distinct melanoma tumors and are thus true melanoma cells. Cell lines were not further authenticated

Mycoplasma contamination

Cell lines tested negative for mycoplasma infection and were subsequently treated with Plasmocin to prevent contamination

Commonly misidentified lines
(See ICLAC register)

No commonly misidentified cell lines were used

Animals and other organisms

Policy information about studies involving animals; ARRIVE guidelines recommended for reporting animal research

Laboratory animals

C57BL/6J (JAX stock #000664), OT-I (JAX stock #003831), Rag1-/- (JAX stock #002216) and CD8-/- (JAX stock #002665) mice were obtained from Jackson labs. Foxp3RFP (JAX stock #008374) were a kind gift from K. Ley (LJI). Age (6-12 weeks) and sex-matched mice were used for all experiments. The housing temperature is controlled, ranging from 69-75F, humidity is monitored but not controlled and ranges from 30-70%. The light/dark cycles are from 6am-6pm, respectively. All animal work was approved by the relevant La Jolla institute for Immunology Institutional Animal Care and Use Committee.

Wild animals

No wild animals were used

Field-collected samples

No Field-collected samples were used

Ethics oversight

All animal work was approved by the relevant LJI Animal Ethics Committee

Note that full information on the approval of the study protocol must also be provided in the manuscript.

Human research participants

Policy information about studies involving human research participants

Population characteristics

Newly diagnosed, untreated patients with histologically confirmed HNSCC were prospectively recruited. All patients had tissue collected as a dedicated research biopsy after consent and prior to randomization, with an additional sample collected during surgical resection. All patient characteristics can be found in the Source Data _Patient characteristics

Recruitment

Patients were recruited at four institutions in the UK (University Hospital Southampton NHS Foundation Trust, Poole Hospitals NHS Foundation Trust, Liverpool University Hospitals NHS Foundation Trust and Queen Elizabeth University Hospital Glasgow) and written informed consent was obtained from all subjects. Patients were randomly assigned to either a placebo group or a drug treatment-group. Detailed information about the trial design, randomization procedure, protocol amendments, recruitment data, patient characteristics and adverse events are deposited at https://www.clinicaltrialsregister.eu/ctr-search/trial/2014-004388-20/results#moreInformationSection. Patients were recruited after initial diagnosis and before definitive surgical treatment; drug treatment or placebo was given for up to 24 or 28 days respectively, prior to resection of tumor

Ethics oversight	The study was sponsored by Cancer Research UK Center for Drug Development (CRUKD/15/004) and approved by the Southampton and South West Hampshire Research Ethics Board; the trial EudraCT number is 2014-004388-20

Note that full information on the approval of the study protocol must also be provided in the manuscript.

Clinical data

Policy information about clinical studies

All manuscripts should comply with the ICMJE guidelines for publication of clinical research and a completed CONSORT checklist must be included with all submissions.

Clinical trial registration	https://www.clinicaltrialsregister.eu/ctr-search/trial/2014-004388-20/results
Study protocol	Summary data, including toxicity listings and a list of protocol amendments have been reported on the EU clinical trials register
Data collection	Patients were recruited from October 2015 to May 2018 at in the UK (University Hospital Southampton NHS Foundation Trust, Poole Hospitals NHS Foundation Trust, Liverpool University Hospitals NHS Foundation Trust and Queen Elizabeth University Hospital Glasgow, two additional centers did not recruit patients); written informed consent was obtained from all subjects. Patients were eligible if they were ≥18 years, with histologically proven HNSCC for whom surgery was the primary treatment option, with laboratory results within specified ranges. Patients had to be clinically eligible for tumor resection; patients who had undergone prior radio/immuno/chemotherapy or other anti-cancer therapy for their current HNSCC, were excluded. Clinical data were obtained for age, gender, tumour size (T stage), and nodal status (N stage) (summarised in Source Data_Patient characteristics)
Outcomes	Primary endpoints were safety and assessment of CD8+ immune infiltrates, secondary endpoints tumor responses and AMG319 pharmacokinetic evaluation (https://www.clinicaltrialsregister.eu/ctr-search/trial/2014-004388-20/results#endPointsSection).

Flow Cytometry

Plots

Confirm that:

☒ The axis labels state the marker and fluorochrome used (e.g. CD4-FITC).

☒ The axis scales are clearly visible. Include numbers along axes only for bottom left plot of group (a 'group' is an analysis of identical markers).

☒ All plots are contour plots with outliers or pseudocolor plots.

☒ A numerical value for number of cells or percentage (with statistics) is provided.

Methodology

Sample preparation	Murine samples - Lymphocytes were isolated from spleen by mechanical dispersion through a 70-µm cell strainer (Miltenyi) to generate single-cell suspensions. RBC lysis (Biolegend) was performed to remove red blood cells. Tumor samples were harvested and lymphocytes were isolated by dispersing the tumor tissue in 2ml of PBS, followed by incubation of samples at 37°C for 15min with 800U/ml DNase I (Sigma) and 0.15WU/ml Liberase DL (Roche). Solutions were then diluted with MACS buffer and grinded through a 70-µm cell strainer to generate a single cell solution<br><br>Human samples - Human TILs were isolated from cryopreserved tumor tissue using a combination of enzymatic and mechanical dissociation. TILs were isolated by dispersing the tumor tissue in 1ml of PBS, followed by incubation of samples at 37°C for 15min with 800U/ml DNase I (Sigma) and 0.15WU/ml Liberase DL (Roche). Solutions were then diluted with MACS buffer and grinded through a 70-µm cell strainer to generate a single cell solution
Instrument	BD LSRFortessa, BD FACSAria-4 Fusion
Software	BD FACSDiva 9<br>FlowJo v10.4.1
Cell population abundance	Sorting efficiency was observed during sorting
Gating strategy	Gating strategy provided in ED Fig. 2 and ED Fig. 3.

☒ Tick this box to confirm that a figure exemplifying the gating strategy is provided in the Supplementary Information.
